# Prognostic significance of cyclin D1 protein expression and gene amplification in invasive breast carcinoma

**DOI:** 10.1371/journal.pone.0188068

**Published:** 2017-11-15

**Authors:** Angela B. Ortiz, Diego Garcia, Yolanda Vicente, Magda Palka, Carmen Bellas, Paloma Martin

**Affiliations:** 1 Pathology Department, Instituto de Investigación Sanitaria Puerta de Hierro-Majadahonda (IDIPHIM) Majadahonda, Madrid, Spain; 2 Medical Oncology Department, Instituto de Investigación Sanitaria Puerta de Hierro-Majadahonda (IDIPHIM) Majadahonda, Madrid, Spain; 3 Centro de Investigación Biomédica en Red de Cáncer (CIBERONC), Madrid, Spain; University of South Alabama Mitchell Cancer Institute, UNITED STATES

## Abstract

The oncogenic capacity of cyclin D1 has long been established in breast cancer. *CCND1* amplification has been identified in a subset of patients with poor prognosis, but there are conflicting data regarding the predictive value of cyclin D1 protein overexpression. This study was designed to analyze the expression of cyclin D1 and its correlation with *CCND1* amplification and their prognostic implications in invasive breast cancer. By using the tissue microarray technique, we performed an immunohistochemical study of ER, PR, HER2, p53, cyclin D1, Ki67 and p16 in 179 invasive breast carcinoma cases. The FISH method was performed to detect *HER2/Neu* and *CCND1* amplification. High cyclin D1 expression was identified in 94/179 (52%) of invasive breast cancers. Cyclin D1 overexpression and *CCND1* amplification were significantly associated (*p* = 0.010). Overexpression of cyclin D1 correlated with ER expression, PR expression and Luminal subtypes (*p*<0.001), with a favorable impact on overall survival in the whole series. However, in the Luminal A group, high expression of cyclin D1 correlated with shorter disease-free survival, suggesting that the prognostic role of cyclin D1 depends on the molecular subtype. *CCND1* gene amplification was detected in 17 cases (9%) and correlated significantly with high tumor grade (*p* = 0.038), high Ki-67 protein expression (*p* = 0.002), and the Luminal B subtype (*p* = 0.002). Patients with tumors with high amplification of *CCND1* had an increased risk of recurrence (HR = 2.5; 95% CI, 1.2–4.9, *p* = 0.01). These findings suggest that *CCND1* amplification could be useful for predicting recurrence in invasive breast cancer.

## Introduction

Invasive breast cancer (IBC) is one of the leading causes of mortality in women worldwide [1]. Many investigative efforts have focused on a better understanding of IBC’s oncogenic pathways and the search for new breast cancer biomarkers, of prognostic and therapeutic predictive value. The expression of estrogen receptor (ER), progesterone receptor (PR) and HER2, and the identification of molecular subtypes (Luminal A, Luminal B, HER2 enriched and Basal like) have important prognostic and predictive roles in the clinical management of IBC [2,3]. However, there are many other biomarkers that are related to the progression and therapeutic response of IBC, but a lack of consistent results in different studies has limited their use in clinical practice.

The cyclin D1 and cyclin-dependent kinase 4 and 6 (CDK4/6) complex pathway is involved in cell cycle regulation and several downstream signals. During cell cycle progression, the cyclin D1-CDK4/6 complex mediates the phosphorylation and inactivation of the retinoblastoma protein (pRb), allowing cells to progress from G1 phase to S phase [4]. Dysregulation of the CDK4/6- cyclin D1 complex is an important step in the genesis of breast cancer, and several genetic alterations in cell cycle regulatory proteins have been described. Cyclin D1 also has CDK-independent functions and may activate ER-mediated transcription independently of estrogen and thereby potentially modify the estrogen response [5]. p16INK4a (p16) acts as a CDK inhibitor by inactivating CDK4/6 and preventing the phosphorylation of Rb. Inactivation of p16 causes unregulated persistent Rb phosphorylation, resulting in loss of control of cell cycle arrest. Furthermore, cyclin D1 may act through CDK-independent pathways. Cyclin D1 interacts with a variety of other transcription factors, including estrogen receptor (ER), androgen receptor, histone deacetylases and acetylases, suggesting that cyclin D1 plays an important role in the regulation of transcription, in addition to its CDK-dependent function in cell cycle progression [6,7].

Cyclin D1 dysregulation in human breast cancer cells *in vitro* promotes progression to G1⁄S transition, with loss of growth control, decreasing the dependence of these cells on growth factors [8]. By contrast, in normal human mammary epithelial cells, cyclin D1 overexpression causes growth inhibition rather than growth, induces differentiation, and enhances apoptosis. By contrast, cyclin D1 overexpression in transgenic mammary tissues results in mammary hyperplasia and tumors [9].

Overexpression of cyclin D1 is observed in approximately 50% of IBC [10,11], and 5% to 20% of these tumors have *CCND1* gene amplification. Some groups have reported that cyclin D1 overexpression is a predictor of worse prognosis [12,13], while others have found an association with an ER-positive phenotype and a better clinical outcome [11,14,15]. However, a few ER-negative tumors express cyclin D1, demonstrating that this protein can also have an oncogenic role in hormone-independent breast carcinoma pathways. High *CCND1* gene amplification is related to an aggressive tumor behavior and poor prognosis [16].

In the present work, we assessed the expression of cyclin D1, amplification of the *CCND1* gene and p16 expression in IBC samples, correlated the findings with known prognostic factors and investigated the correlations of these three markers with survival functions.

## Materials and methods

### Patients and samples studied

A total of 188 cases of partial and total mastectomies from 2002 to 2012 were selected from a database of the Department of Pathology of Hospital Universitario Puerta de Hierro-Majadahonda, Madrid. Hematoxylin-eosin (HE) slides were reviewed by two pathologists (ABOO and CBM) and classified according to TNM stage. A paraffin block of each case with a representative tumor sample was selected and included in the tissue microarrays. Two cores with a diameter of 1 mm were punched out from viable morphologically representative areas of each paraffin block of the selected samples using the Tissue Arrayer device (Beecher Instrument, Silver Spring, MD, USA).

In addition, the pathology and medical records were reviewed. Clinicopathological information, including patient age, tumor size, lymph node status, local recurrence, distant metastasis, and survival, was collected. Nine cases were excluded from the study due to a lack of sample. A total of 179 IBC cases were finally included in this study.

#### Ethics statement

The study was carried out in accordance with Good Clinical Practice guidelines and applicable regulations, as well as the ethical principles originating in the Declaration of Helsinki. The protocol was reviewed and approved by the Ethics Committee of Hospital Universitario Puerta de Hierro-Majadahonda (Acta n° 240, 26/01/09). The review board approved waiver of the requirement to obtain informed consent because the research design involved no more than minimal risk and a requirement of individual informed consent would make the conduct of the research impracticable. Patient information was anonymized prior to analysis.

### Immunohistochemical staining

Sections of 3 μm of the TMA paraffin blocks were processed for IHC. A total of 7 antibodies were used for IHC analysis (Table 1). For ER, PR, HER2, p53, cyclin D1 and Ki67 the staining procedures were performed on the Autostainer Link48 system (DAKO, California, USA). The LeicaBond III system (Leica Microsystems, Wetzlar, Germany) was used for the IHC study of p16 according to the manufacturer’s instructions. Normal breast tissue was used as a control for immunohistochemistry.

**Table 1:** Panel of antibodies used in this study.

Antibody	Clone	Source	Dilution
ER	1D5	DAKO	1:200
PR	PGR636	DAKO	1:200
Ki-67	MIB-1	DAKO	1:50
Her2	POLYCLONAL	DAKO	PREDILUTED
Cyclin D1	SP4	DAKO	PREDILUTED
p16	EGH4	MTM	PREDILUTED
p53	DO-7	NOVOCASTRA	1:50

ER = estrogen receptor, PR = progesterone receptor, Her2 = human epidermal growth factor receptor 2

ER and PR positivity were defined as nuclear staining in 1% or more of tumor cells [17]. Staining of cyclin D1 was assessed as the fraction of nuclear staining fraction cells: 0, <10%, 10–50%, >50%. For p16, nuclear and cytoplasmic staining was regarded and scored according to the percentage of cell staining <10%, 10–50%, and >50%. HER2 staining was analyzed according to the American Society of Clinical Oncology and College of American Pathologists guidelines [17]. HER2 immunostaining was considered positive in specimens that received a score of 3+, whereas scores of 0 to 1+ were regarded as negative. Cases given a score of 2+ were classified as borderline. Ki-67 status was scored low if 14% or less of the nuclei of the neoplastic cells were positive and high if ≥15% of the nuclei of tumor cells were positive. The cut-off value of p53 positivity was staining of greater than 25% of tumor cells [18].

### Fluorescence in situ hybridization

Interphase FISH (fluorescent in situ hybridization) analysis was performed on 3-μm TMA tissue sections using commercial probes. *CCND1* amplification was assessed using LSI *Cyclin D1* (11q13) SpectrumOrange/CEP11 SpectrumGreen probe (Abbott Molecular, Abbott Park, Illinois, USA) according to previously described methods [19].

*HER2* FISH was performed using the LSI HER2/CEP17 probe (Leica BOND) in the Leica BOND-III system according to the manufacturer’s instructions. The results of *HER2* FISH were classified following ASCO/CAP recommendations for the assessment of HER2.

All samples were evaluated by two investigators (ABOO and PMA) using a Leica DM 5000B fluorescence microscope. The assessment was made in 2 tumoral areas with at least 25 neoplastic cells. For *CCND1* evaluation, high-level gene amplification was defined as more than 10 copies per nucleus or high copy clusters in >50% of the cells [20]. Low-level amplification was defined as 6–10 copies in >50% of cells [21]. Samples with 1–5 copies were classified as non-amplified.

### Statistical analysis

Statistical analysis was performed using SPSS 15.0 for Windows (SPSS Inc., Chicago, IL, USA). Categorical variables were analyzed with the Chi square test and, if necessary, Fisher’s exact test. Two-tailed *p* values of less than 0.05 were considered to indicate statistical significance. Survival curves were calculated according to the Kaplan-Meier method using Cox regression, and differences between curves were evaluated using the log-rank test. Overall survival (OS) was calculated from the date of diagnosis to the date of death or last follow-up. Disease-free survival (DFS) was defined as the time from diagnosis to first locoregional recurrence, distant recurrence or contralateral disease.

## Results

### Clinicopathological data

All 179 patients were women, and their median age was 57 years (range 28 to 93 years). The clinical and pathological features are described in Table 2. The majority of the tumors (96 cases, 54%) were tumor stage T2, 70 cases (39%) were T1, and tumors greater than 5 cm (T3) were observed in only 13 cases (7%). The distribution of the tumors in the study by Nottingham histological grade showed that 31 cases (17%) were histological grade 1, 66 cases (37%) were grade 2 and 82 cases (46%) were grade 3. Eighty-six patients had metastatic lymph nodes, and 86 had no lymph nodes compromised by tumors. In 7 cases, there was no lymph node biopsy. Classification according to IHC revealed the following: 68 (38%), 63 (35%), 13 (7%), and 35 (20%) lesions were classified as Luminal A, Luminal B, HER2, and Triple Negative, respectively (Table 2). Follow-up data were available in 158 cases; recurrence occurred in 49 cases, while 30 patients died of the disease.

**Table 2:** Patients’ characteristics (n = 179).

Characteristic	No. (%)
Age (yr)	
Median	57
Range	28–93
Tumor size (cm)	
≤ 2	70 (39)
2,1–5	96 (54)
> 5	13 (7)
Tumor grade	
Grade 1	31 (17)
Grade 2	66 (37)
Grade 3	82 (46)
Lymph nodes	
N0	86 (48)
N1	48 (27)
N2	26 (14)
N3	12 (7)
Unknown	7 (4)
Breast Cancer subtypes	
Luminal A	68 (38)
Luminal B	63 (35)
HER2	13 (7)
Triple Negative	35 (20)

### IHC and FISH results

**Fig 1:**
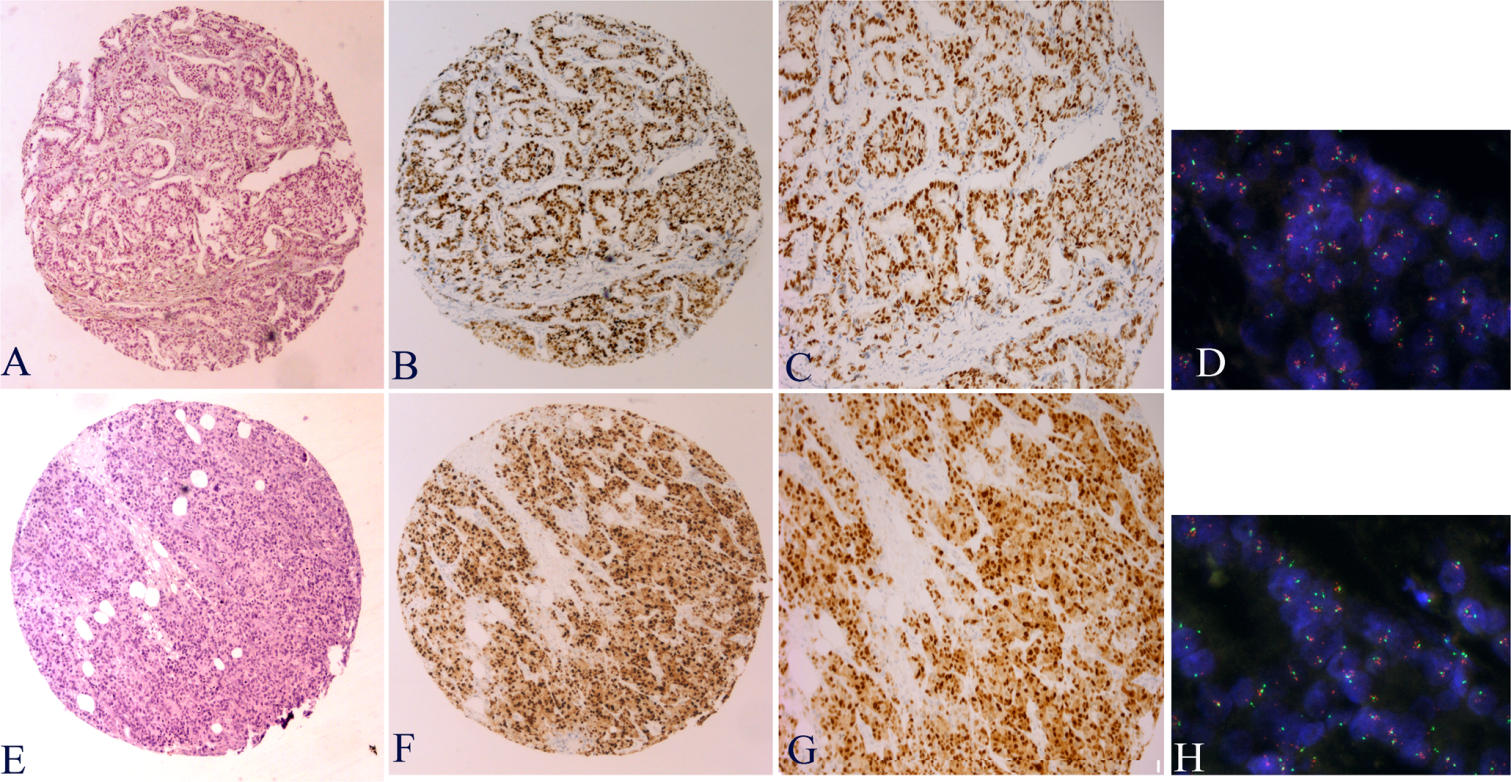
Immunohistochemistry staining for cyclin D1 and *CCND1* amplification (FISH) in invasive ductal breast carcinoma. Examples of cyclin D1 expression: B-C strong nuclear staining in >50% of cells. F-G nuclear and cytoplasmic staining in most nuclei of invasive breast carcinoma. *CCND1* gene amplification was evaluated by FISH. D- Clusters of *CCND1* amplification, H- Low-level *CCND1* amplification.

Cyclin D1 protein nuclear staining was observed in more than 50% of cells in 94 cases (52%), between 10–50% of cells in 52 cases (29%), and <10% in 21 cases (12%) and was negative in 12 cases (7%) (Fig 1). The IHC positivity of cyclin D1 correlated with the expression of ER (*p*<0,001) and PR (*p*<0,001) as well as with Luminal type (*p*<0,001). Loss of cyclin D1 expression correlated with high histological grade (*p* = 0,008), high Ki67 expression (*p* = 0,026), p16 expression (*p*<0,001) and Triple Negative subtype (*p*<0,001). Although most of cases cyclin D1 positive has been part to Luminal type, high expression of cyclin D1 was found in a small number of Triple Negative tumors (4 of 35 cases) and HER2-enriched cases (4 of 13). No statistical correlation with p53 expression was observed.

*CCND1* amplification was detected in 34 cases with two different patterns: high amplification was observed in 17 cases (9%), with more than 10 signals per nucleus, whereas the other 17 showed low amplification. For the remaining 145 tumors (81%) no amplification of *CCND1* was observed by FISH. *CCND1* amplification correlated significantly with higher tumor grade (*p* = 0.038), high Ki-67 protein expression (*p* = 0.002), and Luminal B subtype (*p* = 0.002): 13 of 17 cases (76.5%) with high amplification were the Luminal B subtype (Table 3). No statistical correlations were found with tumor size, lymph node involvement, ER, PR, *HER2* amplification, p53 or p16.

**Table 3:** Cyclin D1 expression and *CCND1* amplification in breast cancer subtypes.

Subtype	Cyclin D1 Negative	Cyclin D1 <10%	Cyclin D1 10–50%	Cyclin D1 >50%	*CCND1* Not A	*CCND1* low A	*CCND1* high A
Luminal A	0	5 (7.3%)	22 (32.4%)	41 (60.3%)	56 (82.3%)	11 (16.2%)	1(1.5%)
Luminal B	2 (3.2%)	1 (1.6%)	15 (23.8%)	45 (71.4%)	45 (71.4%)	5 (7.9%)	13 (20.7%)
HER2	0	4 (30.8%)	5 (38.5%)	4 (30.8%)	12 (92.3%)	0	1(7.7%)
Triple Negative	10 (28.6%)	11 (31.4%)	10 (28.6%)	4 (11.4%)	32 (91.4%)	1 (2.9%)	2 (5.7%)

Not A: not amplified, low A: amplification ≤10 signals per nuclei, high A: amplification >10 signals

Cyclin D1 protein overexpression and *CCND1* amplification were significantly associated (*p* = 0.010); all cases with high amplification (17/17) and 15/17 cases with low amplification showed nuclear protein expression. All cases that were cyclin D1 negative by immunohistochemistry and most cases (19/21) with low expression (<10%) showed no amplification signals. In the non-amplified group, 45% (66/145) of cases showed cyclin D1 expression in >50% of cells (Table 4).

**Table 4:** Correlation between FISH status and cyclin D1 immunohistochemistry results.

	No amplification	Low amplification	High amplification	
IHC Negative	12	0	0	12
IHC <10%	19	2	0	21
IHC 10–50%	48	2	2	52
IHC >50%	66	13	15	94
*p* = 0.010	145	17	17	179

Regarding other markers, nuclear p16 expression was observed in 69% (124) of the breast carcinoma specimens: 62 cases with expression between 10–50% of cells and 62 cases in more than 50% of cells. Nuclear p53 expression was observed in 84 cases (47%) with the following distribution: 21 cases in the Luminal A type group, 30 in the Luminal B group, 6 in the HER2 group and 27 cases in the Triple Negative group. Ninety-three samples (52%) showed a high proliferation index (Ki-67>15%). The *HER2* gene (FISH) was non-amplified in 152 cases (85%) and amplified in 27 cases (15%).

We observed a correlation between p53 positivity and Triple Negative subtype (*p*<0.001); 77% of these cases (27 of 35) showed p53 expression. Of the 35 Triple Negative tumors, 27 (77%) showed high Ki-67 expression. Most ER negative cases were p16 positive, with staining in >50% of cells (*p*<0,001), similar to the PR negative cases (*p* = 0,003). P16 immunopositivity was observed in 27 of 35 Triple Negative tumors (*p* = 0,001) and correlated with high Ki-67 levels (*p* = 0.039) and high tumor grade (*p* = 0.003). No significant association was observed between p16 expression and *HER2*, p53, tumor size or nodal infiltration.

### Prognostic significance of cyclin D1 expression and *CCND1* amplification

**Fig 2:**
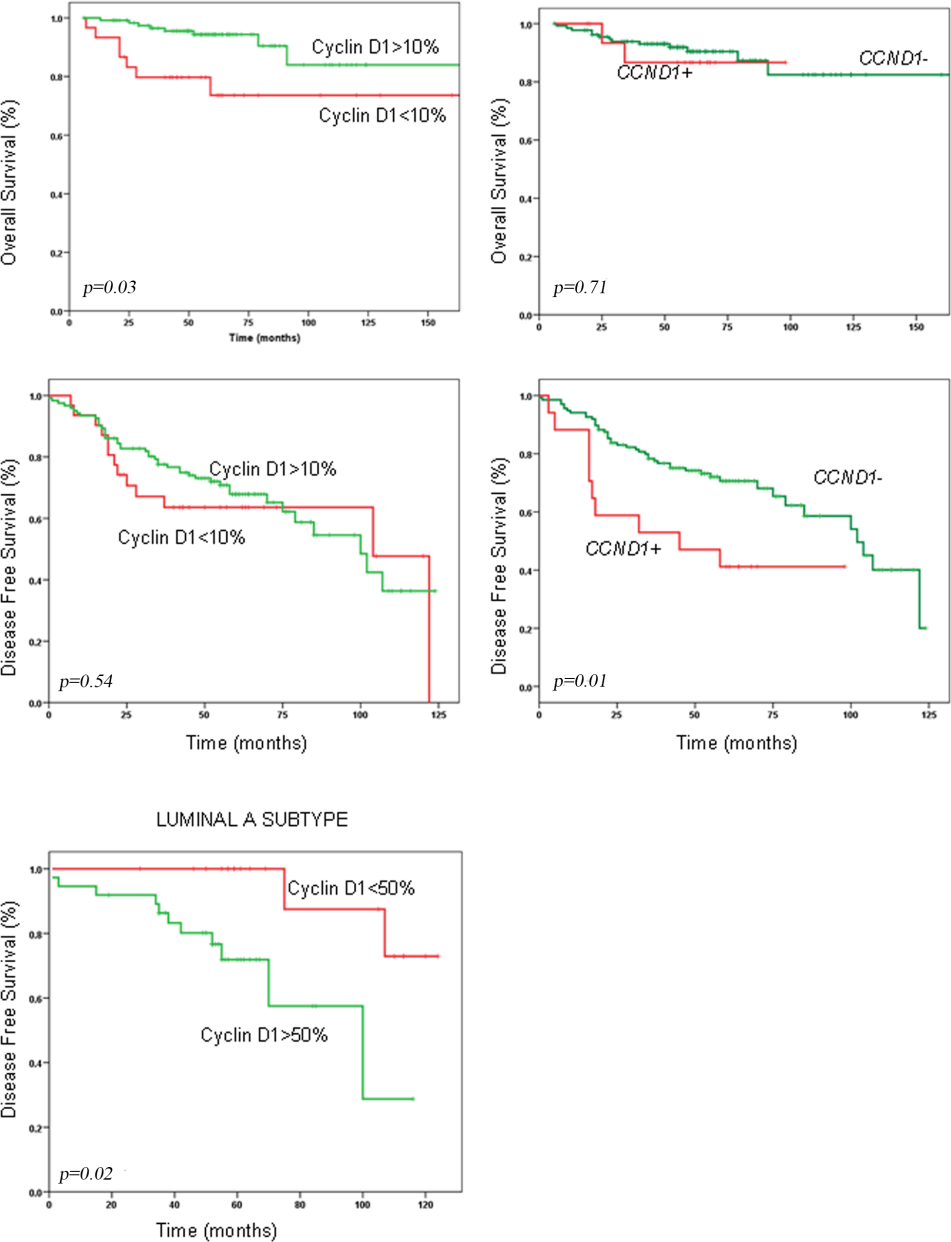
Kaplan-Meier survival analysis revealed that the expression of cyclin D1was predictive for overall survival (OS) but not disease-free survival (DFS). *CCND1* amplification was predictive for DFS. High cyclin D1 expression affected DFS in Luminal A cases.

Cyclin D1 overexpression was found to have a favorable impact on overall survival (OS) (HR = 0.33; 95% CI, 0.12–0.89, *p* = 0.03) but had no significant association with disease-free survival (DFS) considering the whole series (Table 5) (Fig 2). When we analyzed molecular subtypes individually, high expression of cyclin D1 protein (>50% of nuclei) correlated significantly with shorter DFS (*p* = 0.029) in Luminal A cases. Patients with tumors with high amplification of *CCND1* (>10 copies) had an increased risk of recurrence compared with patients with no amplification or low amplification in tumors (HR = 2.5; 95% CI, 1.2–4.9, *p* = 0.01). In the whole series, there was no significant difference in OS between cases with *CCND1* amplification and cases without amplification. When we analyzed separately the prognostic implication of *CCND1* amplification in RE-positive and -negative tumors, we found that high amplification conferred worse OS in RE-positive cases (*p =* 0.026), but not RE-negative cases.

**Table 5:** Univariate Cox regression analysis for clinicopathological factors.

	Overall survival			Disease-free survival		
	Hazard ratio	95%CI	*p*	Hazard ratio	95%CI	*p*
Tumor size	3.20	1.74–5.90	0.008	2.05	1.31–3.20	0.002
Grade	2.04	1.12–3.72	0.019	1.59	1.07–2.36	0.021
Lymph node	2.69	1.44–5.03	0.002	2.15	1.31–3.52	0.002
ER expression	0.31	0.15–0.63	0.001	0.40	0.24–0.68	0.001
PR expression	0.39	0.19–0.81	0.012	0.43	0.26–0.74	0.002
*HER2* amplification	2.10	0.96–4.59	0.064	2.16	1.15–4.04	0.016
P53 expression	3.50	1.13–10.88	0.019	1.08	0.64–1.85	0.764
P16 expression	1.41	0.89–2.21	0.140	1.14	0.82–1.59	0.418
*CCND1* amplification	1.09	0.64–1.84	0.753	2.47	1.24–4.96	0.010
Cyclin D1 expression	0.33	0.12–0.89	0.029	0.825	0.44–1.54	0.547

Molecular subtype classification correlated with OS and DFS (HR = 1.7; 95% CI, 1.2–2.7, *p* = 0.006 and HR = 1.4; 95% CI, 1.1–1.8, *p* = 0.02 respectively). No statistically significant correlation was found between p16 expression and any of the survival functions. Cases with p53-positive tumors had shorter OS (HR = 3.5; 95% CI, 1.13–10.88, *p* = 0.019) than negative cases.

In multivariate analysis tumor size, lymph node, *CCND1* amplification and p53 expression were associated with shorter OS, and cyclin D1 expression (>10%) was associated with longer OS (Table 6). Tumor size, lymph node, and *CCND1* amplification were associated with shorter DFS.

**Table 6:** Cox multivariate analysis for prognostic factors.

	Overall survival			Disease-free survival		
	Hazard ratio	95%CI	*p*	Hazard ratio	95%CI	*p*
Tumor size	5.10	1.64–15.83	0.005	1.86	1.12.-3.06	0.016
Lymph node	6.86	2.15–21.87	0.001	2.08	1.20–3.60	0.009
P53 expression	5.97	1.62–21.92	0.014	1.29	0.74–2.25	ns
*CCND1* amplification	4.65	0.82–26.37	ns	3.03	1.47–6.27	0.003
Cyclin D1 expression	0.12	0.035–0.44	0.001	0.57	0.29–1.12	ns

## Discussion

The prognostic and predictive value of cyclin D1 overexpression in breast cancer remains controversial. Cyclin D1 protein expression has been reported to be a prognostic marker in breast carcinoma, and most studies have shown that overexpression is a good prognostic factor, particularly for ER-positive patients [14,15,22]. However other authors [12,13] have reported that cyclin D1 overexpression is a predictor of poor prognosis; cyclin D1 promotes the phosphorylation of retinoblastoma protein (Rb) and other substrates by binding to cyclin-dependent kinase 4/6 (CDK4/6) to make cells proliferation rapidly.

Detection of overexpression of cyclin D1 by immunohistochemistry has been reported in 35–81% of breast carcinomas, in line with our results. In our series, 52% of cases were cyclin D1 positive in more than 50% of cells. We observed a positive correlation between a lack of cyclin D1 expression and tumor grade and proliferation, which confers an aggressive course of disease. Cyclin D1 plays a crucial role as a cell cycle regulator, promoting progression through G1-S phase [14], and thus one would expect that enhancement of cyclin D1 expression would be associated with larger tumors with higher proliferation rates [23], however, expression of cyclin D1 is associated with lower histological grade [11,21,24,25]. Mylona *et al* [23] proposed an interaction between cyclin D1, histone acetylases and Rb such that acetylation of Rb leads to cell cycle exit and induces growth arrest.

Cyclin D1 expression has been reported to correlate with ER expression [26], in agreement with our findings that cyclin D1 overexpression correlates with ER and PR expression and with Luminal subtypes. This results supports the critical role of cyclin D1 in estrogen-induced breast cancer, as estrogen action is mediated through transcriptional activation of cyclin D1 and c-Myc [16,22,27].

*CCND1* amplification has been assumed to be critically involved in tumor initiation and progression by proto-oncogene activation. In the present study, 18% of cases had *CCND1* amplification, consistent with previous reports [23,28]. Significant associations were observed between gene amplification and high tumor grade (*p* = 0.038), high Ki-67 protein expression (*p* = 0.002), and Luminal B subtype (*p* = 0.002), all markers of worse prognosis. There was a strong correlation between *CCND1* gene amplification and cyclin D1 protein expression (*p* = 0.010); all cases with >10 gene copies showed overexpression.

Our series confirms that cyclin D1 overexpression (more than 10% of cells) has a favorable impact on OS (HR = 0.33; 95% CI, 0.12–0.89, *p* = 0.029) but not DFS: Luminal A cases with high expression of cyclin D1 protein had shorter DFS (*p* = 0.029). Other studies have reported that cyclin D1 overexpression is significantly associated with longer OS also failed to show a significant difference in DFS [25]. Patients with cyclin D1-overexpressing tumors survive longer but with metastatic disease after recurrence, possibly due to tamoxifen resistance produced by cyclin D1 overexpression [29].

We have found that patients with tumors with high amplification of *CCND1* (>10 copies) had an increased risk of recurrence (HR = 2.5; 95% CI, 1.2–4.9, *p* = 0.01), although there was no difference in OS. This result supports the link between high-level amplification of *CCND1* and worse prognosis [16]. The differences between high and low amplification levels reflect different mechanisms of amplification, as reported for other genes such as *c-MYC* or *HER2*. Only high levels correspond to “true” amplification, whereas low levels appear to be related with polysomy or other chromosomal alterations (duplications or translocations).

Other genes in the 11q13 region, e.g. *MYEOV*, *ORAOV1*, *FGF19*, *FGF4*, *INT2*, *CTTN*, *EMSY*, and *GARP1*, have been identified as driver genes and might be co-amplified with *CCND1* [29–31]. *EMSY* encodes a protein that is linked to the BRCA2 pathway; its activation may function as a surrogate for *BRCA2* loss, conferring a phenotype similar to *BRCA2* tumors. *EMSY* may be a potential predictor of tamoxifen resistance. Moreover, amplification of 11q13 involves a deletion of the q-arm distal to the amplified region; this region harbors a number of genes involved in DNA repair. Interestingly, *CHK1* (11q24) is one of the key regulatory factors of the DNA damage checkpoint, and concurrence of *CHK1* deletion and *CCND1* amplification has been associated with high tumor grade in invasive breast carcinoma [20].

*CCND1-*amplified tumors are a separate entity within RE+, Luminal B subtype and high-grade breast carcinomas, with shorter DFS and poor outcome. Notably, these tumors also over-express cyclin D1; hence immunohistochemical analysis is not useful to identify this aggressive subgroup with poor prognosis.

Recently, Ahlin *et al* reported the association of high cyclin D1 expression with high proliferation and worse prognosis in early breast cancer in ER-positive tumors [32], but not ER-negative cases. When we analyzed ER-positive and ER-negative cases separately, worse OS was found in ER-positive cases with high expression of cyclin D1. In ER-negative cases, no significant association was observed. Accordingly, Lamb *et al* [33] reported that cyclin D1 overexpression increases migration and more aggressive behavior in ER-positive breast cancer cell lines, whereas in ER-negative cell lines the opposite occurs, supporting different effects of cyclin D1 expression in ER-positive and ER-negative cells. In ER negative breast cancer, cell proliferation may be activated thorough deregulation downstream from the pRb-node with overexpression of cyclin E [34]. High cyclin E expression causes chromosomal instability and is often associated with aggressive disease features.

The loss of p16 has frequently been described in several cancers, and its inactivation may contribute to cancer progression and poor prognosis. The role of p16 in human breast cancer remains controversial. An association of ER negativity, high grade and proliferation activity with the overexpression of p16 has been detected in previous breast cancer studies. ER negativity has also been associated with a basal-like phenotype [35]. Consistent with these reports, we observed that p16 overexpression is a marker of high tumor grade and is associated with a lack of cyclin D1 expression and Triple Negative subtype. We observed a relationship between p16 expression and high Ki-67 levels regardless of the status of p53, similar to Sugianto *et al* [36]; however, our study fail to find a role of p16 in proliferation and aggressiveness in p53-negative tumors, probably because only 8 of 35 Triple Negative cases in our series were p53 negative. Although p16 expression appeared to be a marker of aggressive morphology in our series we did not observed an association with any of the survival functions. Several discrepancies in the role of p16 in breast cancer survival have been described; some authors have reported a significant association between high p16 expression and increased breast cancer-specific survival and DFS [22], whereas others have associated p16 expression with poor survival [37].

In summary, our results indicate that cyclin D1 overexpression is associated with types of breast cancer with good prognostic features (ER, PR, low grade), but appears to play different prognostic roles in different molecular subtypes. *CCND1* gene amplification is predictive of shorter DFS and poor outcome. The discrepancies in the prognostic value of the protein and the gene may be attributable to the distinct functions of cyclin D1 in different cell cycle phases; the mechanism is complex and not mediated by a single pathway or gene product [28]. Expression of cyclin D1 is regulated at the transcriptional level by several pathways; activated Ras promotes transcription of *CCND1* thorough a kinase pathway involving Raf1, MAPK and ERKs. PTEN blocks cell cycle progression through downregulation of cyclin D1. Under some conditions, cyclin D1 may induce growth arrest instead of cell cycle progression [23], and therefore we suggest that protein expression should be analyzed in the context of molecular subtypes.
